# RDH10 Oxidation of Vitamin A Is a Critical Control Step in Synthesis of Retinoic Acid during Mouse Embryogenesis

**DOI:** 10.1371/journal.pone.0030698

**Published:** 2012-02-02

**Authors:** Lisa L. Sandell, Megan L. Lynn, Kimberly E. Inman, William McDowell, Paul A. Trainor

**Affiliations:** 1 Stowers Institute for Medical Research, Kansas City, Missouri, United States of America; 2 Department of Molecular, Cellular and Craniofacial Biology, University of Louisville, Louisville, Kentucky, United States of America; 3 Department of Anatomy and Cell Biology, University of Kansas Medical Center, Kansas City, Kansas, United States of America; Instituto de Medicina Molecular, Portugal

## Abstract

Retinoic Acid (RA) is a small lipophilic signaling molecule essential for embryonic development and adult tissue maintenance. Both an excess of RA and a deficiency of RA can cause pathogenic anomalies, hence it is critical to understand the mechanisms controlling the spatial and temporal distribution of RA. However, our current understanding of these processes remains incomplete. Vitamin A is metabolized to RA via two sequential enzymatic reactions. The first requires retinol dehydrogenase (RDH) activity to oxidize Vitamin A (retinol) to retinal, and the second requires retinaldehyde activity (RALDH) to oxidize retinal into RA. The first reaction has previously been attributed to the alcohol dehydrogenase (ADH) family, whose genes are ubiquitously or redundantly expressed. Consequently, the specificity of RA synthesis was thought to reside exclusively at the level of the second reaction. To better understand the metabolism of Vitamin A into RA during embryogenesis, we generated new mouse models that disrupt this process. Here we describe a new targeted knockout of *Rdh10* in which RA synthesis is severely impaired, particularly at critical early embryonic stages. We also introduce a new mutant allele of *Aldh1a2*. Both mutations produce similar developmental defects resulting in lethality around embryonic day 10.5 (E10.5). The severity of the *Rdh10* null phenotype demonstrates that embryonic oxidation of retinol is carried out primarily by RDH10 and that neither ADHs nor other enzymes contribute significantly to this reaction. We also show that reduced RA production results in upregulation of *Rdh10.* These data demonstrate that RDH10 plays a critical role in mediating the rate limiting RDH step of Vitamin A metabolism and functions as a nodal point in feedback regulation of RA synthesis. Moreover, RDH10-mediated oxidation of retinol plays as important a role in the control and regulation of RA production during embryogenesis as does the subsequent RALDH-mediated reaction.

## Introduction

Retinoic acid (RA) is a derivative of Vitamin A (retinol) that plays an essential role in many vertebrate biological processes including energy metabolism, brain function, immune response, reproduction and embryonic development (reviewed in [1], [2], [3], [4], [5], [6]). The processes of embryonic growth and patterning are particularly dependent on this small molecule, as can be appreciated by the striking abnormalities and midgestation lethality that occur when RA metabolism is limited or disrupted during development in mice [7], [8], [9], [10] or other mammals [11], [12], [13], [14]. The defects arising from RA perturbation occur because RA controls the transcription of a variety of essential developmental genes by virtue of its function as a ligand that binds to retinoic acid receptors (RAR). The RAR, which form heterodimers with retinoid receptors (RXR), activate or repress gene activity when RA is bound (reviewed in [15]). Owing to the importance of RA in regulating essential developmental processes, it is critical to understand the mechanisms that regulate the metabolism of this key compound.

Unlike many signaling molecules whose function is modulated by changes in RNA transcription or protein activity, RA is a small molecule whose level and tissue distribution are regulated by biochemical synthesis and by degradation. It has been known for some time that synthesis of RA from the dietary precursor retinol occurs via two sequential enzymatic reactions - first retinol is oxidized to form retinal and, second, retinal is oxidized to form RA [16]. The first reaction is facilitated by enzymes with retinol dehydrogenase (RDH) activity and the second reaction is carried out by enzymes with retinaldehyde dehydrogenase activity (RALDH).

The RALDH second step of RA synthesis is carried out by RALDH1, RALDH2 and RALDH3, which are encoded by *Aldh1a1*, *Aldh1a2* and *Aldh1a3*, respectively [7], [8], [9], [10]. RALDH2 is the primary enzyme responsible for conversion of retinal to RA during early embryogenesis, mediating the major part of RA synthesis within the early developing embryo [8]. Loss of RALDH2 function eliminates RA signaling within the trunk region and results in severe developmental abnormalities, including shortened anteroposterior axis, defects in embryo turning, dilated and un-looped hearts, small somites, absence of forelimb buds and lethality by embryonic day 10.5 (E10.5). Loss of RALDH3 disrupts RA signaling in the embryonic head, resulting in defects in formation of the nose and eye and lethality at birth. The severe phenotypes of embryos lacking RALDH2 or RALDH3 clearly demonstrate the critical nature of the RALDH reaction in embryonic RA synthesis.

In contrast to the well-established importance of the RALDH second reaction, the RDH first reaction of RA synthesis has received less attention due to the misperception that it occurs ubiquitously throughout embryogenesis. Two types of molecule are capable of RDH activity *in vitro*: members of the alcohol dehydrogenase (ADH) family, which are cytosolic medium chain reductases (MDR), and members of the RDH family, which are membrane-bound short chain dehydrogenase/reductases (SDR) (reviewed in [17]). ADH enzymes have the ability to catalyze the conversion of retinol to retinal *in vitro* [18]. Owing to this biochemical activity of ADH enzymes, together with an overlapping or ubiquitous pattern of *Adh* gene expression during embryogenesis [19], it was initially proposed that the embryonic oxidation of retinol to retinal was carried out by ADH enzymes within the cytosol [20], [21], [22]. However, despite their biochemical activity and their widespread *in vivo* expression pattern, disruption of single and compound *Adh* genes produced no embryonic defects [21], a lack of effect that was speculatively attributed to potential redundancy within the gene family. The failure to identify an *Adh* mutant embryonic phenotype led to the prevailing view that the first step of embryonic Vitamin A metabolism is mediated by ubiquitous or redundant enzymes and plays a minimal role in the spatiotemporal regulation of RA synthesis.

The notion that the initial oxidative conversion of retinol occurs in an unregulated fashion mediated by ADHs has been challenged by recent discoveries highlighting a critical role for the SDR RDH10 in tissue specific synthesis of RA within the vertebrate embryo. The first indication that RDH10 is important for embryonic synthesis of RA came from characterization of mice with a point mutation termed *Rdh10^trex^* [23]. Embryos homozygous for this mutation exhibit a dramatic reduction in RA synthesis and severe defects in embryonic development including limb, craniofacial and organ defects that result in lethality between E10.5–14.5. Further evidence of a role for RDH10 came from *Xenopus*, in which XRDH10 was shown to be critical for RA synthesis and to be controlled by RA negative feedback inhibition [24]. In zebrafish, the *rdh10a* gene was found to be subject to positive and negative feedback regulation by RA, as was, inversely, *dhrs3a*, whose product catalyzes the reverse reaction converting retinal back into retinol [25]. In chick embryos, expression of *Rdh10* is regulated spatially and temporally in a pattern overlapping with the retinol transporter *Stra6*, but does not appear to be regulated by feedback from excessive or reduced levels of RA [26]. These recent findings raise questions of the specific contribution of RDH10 versus ADHs and of the true mechanistic importance of the first oxidation step of Vitamin A metabolism in controlling embryonic RA synthesis.

The embryonic abnormalities and RA reduction observed in *Rdh10^trex^* mutant mice are dramatic, but generally not as severe as those caused by loss of RALDH2. Embryos lacking RALDH2 do not survive past E10.5 [8], [27], while most homozyogous *Rdh10^trex^* embryos remain viable through E10.5–E11.5 and embryos have been identified surviving as late as E14.5 [23], [28]. The residual production of RA and the less severe phenotype of *Rdh10^trex^* embryos compared to *Aldh1a2* mutant embryos indicate that some RDH activity remains intact in the *Rdh10^trex^* embryos, mediated possibly by hypomorphic activity of the *Rdh10^trex^* point mutant enzyme, by other RDH enzymes, or by ADH enzymes. In order to further our understanding of the regulation of RA synthesis during embryonic development, it is important to know which enzymes catalyze the RDH reaction and whether the relevant enzymes are redundant and ubiquitous within the embryo or if they are instead expressed in a regulated fashion that may have bearing on the control of production of this important signaling molecule.

To clarify the enzymatic regulation of Vitamin A metabolism and RA synthesis during embryogenesis, we have generated new mouse models that disrupt this process and comprise a graded series of RA deficiency. Here we describe a novel targeted knockout allele of *Rdh10*. We show that eliminating RDH10 activity results in severe embryonic abnormalities, more extreme than the phenotype of the original *Rdh10^trex^* point mutant. In this report we also introduce a new point mutant allele of *Aldh1a2*, generated in a chemical mutagenesis screen, which disrupts the RALDH reaction and exhibits a phenotype similar to previously described targeted knockout alleles of *Aldh1a2*. Both the *Rdh10* knockout embryos and the *Aldh1a2* point mutant embryos exhibit similar shortened anteroposterior axes, defects in embryo turning, dilated and un-looped hearts, small somites, and forelimb bud agenesis. Each results in embryo death by ∼E10.5. Using the RARElacZ reporter transgene, we show that RA synthesis in *Rdh10* null embryos is severely reduced at E9.5 and is almost completely eliminated at the earlier critical E8.0–E8.5 stage of development. The severity of the *Rdh10* null phenotype indicates that the RDH mediated oxidation of retinol to retinal during Vitamin A metabolism is carried out primarily by RDH10 and that neither ADH family members nor other enzymes contribute significantly to the RDH step of RA synthesis during early embryogenesis. In this report we also find that *Rdh10* gene expression is elevated when RA levels are reduced, providing evidence that *Rdh10* functions as a control point for feedback regulation of the RA synthesis pathway in mammals. These data demonstrate that the initial RDH reaction of Vitamin A metabolism, mediated primarily by RDH10, plays as critical a role in embryogenesis and is as important in the control and regulation of RA production as is the subsequent RALDH reaction.

## Results

### Graded retinoid deficiency phenotypes: *Rdh10^trex^* vs. *Aldh1a2^gri^*

**Figure 1:**
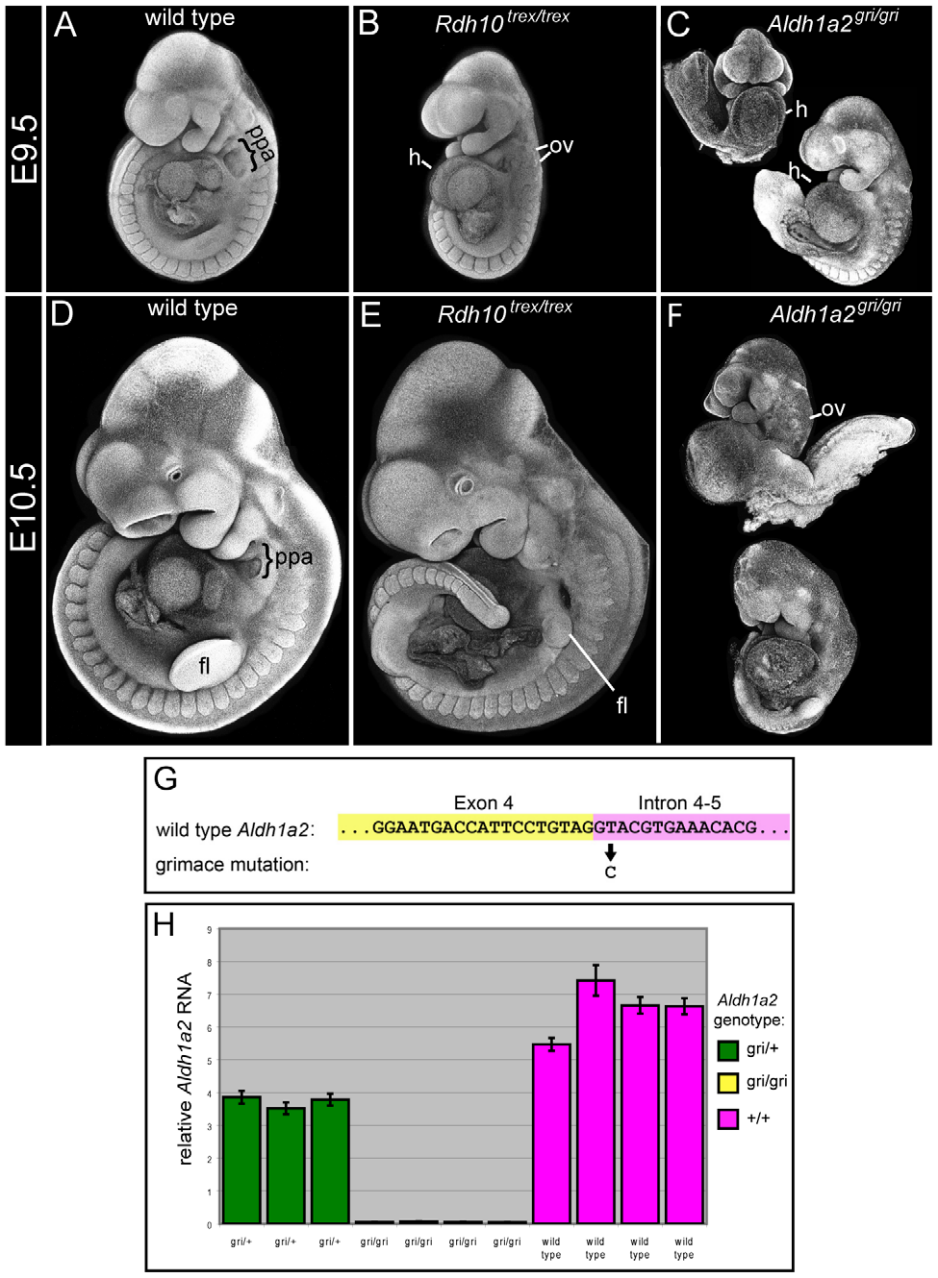
*Rdh10^trex^* and new *Aldh1a2^gri^* mutant phenotype comparison. Wild type (A,D), *Rdh10^trex/trex^* mutant (B,E), and *Aldh1a2^gri/gri^* mutant (C,F) embryos were collected at E9.5 (A–C) and E10.5 (D–F). Formalin-fixed embryos were stained with DAPI and imaged by confocal microscopy. For each embryo, a Z-stack of confocal slices was collapsed to form a “pseudo-SEM” image. (A–F) For each mutant phenotype at each indicated stage n>5 embryos. (G) Sequence of *Aldh1a2* gene at junction between Exon 4 and Intron 4–5 with *grimace* mutation single base change indicated beneath. Exon 4 genomic DNA sequence is indicated by yellow highlight, intron 4–5 sequence indicated by pink highlight. (H) Histogram representing relative levels of *Aldh1a2* mRNA from wild-type, heterozygous *Aldh1a2^gri/+^* and homozygous *Aldh1a2^gri/gri^* embryos, as assessed by quantitative RT-PCR, demonstrating loss of *Aldh1a2* transcript in homozygous mutants. fl, forelimb bud; h, heart; ov, otic vesicle; ppa, posterior pharyngeal arches.

The previously reported mouse *Rdh10^trex^* point mutation disrupts RDH10 function and causes numerous embryonic defects culminating in embryo death between E.10.5–14.5 [23]. In *Rdh10^trex/trex^* mutant embryos RA synthesis is reduced but not completely eliminated, as residual RA signaling is detected in the trunk neural tube. This raised the possibility that *Rdh10^trex/trex^* may be a severe hypomorph rather than a complete null. For comparison with other mutants in the present study, we examined *Rdh10^trex/trex^* embryos at E9.5 and E10.5 (Fig. 1A,B,D,E). Although the *Rdh10^trex/trex^* phenotype is somewhat variable at E9.5, all embryos have numerous discernible abnormalities. First and second pharyngeal arches form in most *Rdh10^trex/trex^* mutant embryos, but third and more posterior pharyngeal arches are absent or fused to the second arch (Fig. 1B,E). Otic vesicles are usually small, duplicated and displaced posteriorly (Fig. 1B). Mutant embryos exhibit cardiac abnormalities, including heart edema and looping defects (Fig. 1B). At E10.5 a reduction in size of the forelimb bud and absence of posterior pharyngeal arches are clearly evident (Fig. 1E). This constellation of defects observed in the *Rdh10^trex/trex^* embryos is consistent with retinoid deficiency but is less severe than that observed in embryos in which RALDH2 function has been eliminated.

As part of the same chemical mutagenesis screen that yielded the *Rdh10^trex^* mutation, we also generated a mutant originally named “*grimace*”[29] that exhibited interesting abnormalities in formation of the pharyngeal arches (Fig. 1C). Mapping, cloning and sequence analysis were used to identify the *grimace* mutation, which was determined to be a single base change within the *Aldh1a2* locus. We therefore henceforth refer to this mutation as *Aldh1a2^gri^*. Sequence analysis of genomic DNA reveals that the *Aldh1a2^gri^* mutation alters the 5′ splice consensus sequence at the boundary of exon 4 and intron 4–5, changing the second intronic nucleotide from a conserved T to a C (Fig. 1G). The altered splice consensus sequence disrupts splicing of *Aldh1a2* mRNA and leads to a reduction in *Aldh1a2* transcript level. Quantitative RT-PCR reveals that *Aldh1a2* transcript in homozygous *Aldh1a2^gri/gri^* mutants is reduced to less than 1% of that of wild type embryos (Fig. 1H). We therefore interpret the new *Aldh1a2^gri^* mutation is either a null allele of *Aldh1a2* or an extreme hypomorph. Consistent with the loss of *Aldh1a2* transcript, we observe the phenotype of homozygous *Aldh1a2^gri/gri^* mutants to be very similar to that of established *Aldh1a2* knockout alleles, each exemplifying the abnormalities associated with severe retinoid deficiency (Fig. 1C,F). Mutant *Aldh1a2^gri/gri^* embryos die by E10.5 exhibiting a number of striking defects including cessation of growth at ∼E8.5–E9.0, defects in axial turning, dilated unlooped hearts, small somites and tiny otic vesicles. Whereas established knock alleles of *Aldh1a2* exhibit no axial turning, many *Aldh1a2^gri/gri^* mutants display a slightly milder phenotype with a small degree of embryo turning. The slightly milder turning defect may represent a difference of strain background on the axial turning phenotype or may indicate that the *Aldh1a2^gri^* allele is not an absolute null.

Comparison of the *Rdh10^trex^* versus the *Aldh1a2^gri^* phenotypes illustrates the less extreme defects elicited by *Rdh10^trex^* mutation relative to that of *Aldh1a2^gri^* (compare Fig. 1 E, F). At E9.5 *Aldh1a2^gri/gri^* mutants have extreme defects in extension and turning of the body axis, with some embryos completely unturned and others with an abnormal half-turned “J” shaped body axis when viewed from the front. In contrast, nearly all E9.5 *Rdh10^trex/trex^*embryos are completely turned. *Aldh1a2^gri/gri^* mutant embryos lack all pharyngeal arches posterior to the first arch, whereas many *Rdh10^trex/trex^* embryos form second arches and, in some cases, rudimentary third arches. The milder phenotype of the *Rdh10^trex/trex^* embryos relative to the severe phenotype of *Aldh1a2^−/−^* mutants, together with the presence of residual RA activity detected using *RARElacZ* reporter mice [23], implies the presence of residual RDH function within *Rdh10^trex/trex^* embryos. The residual RA in these embryos could result from a small amount of RDH activity remaining for the mutant RDH10 protein, or to the function of unidentified RDH or ADH enzymes.

### Generation of *Rdh10* knockout mice

**Figure 2:**
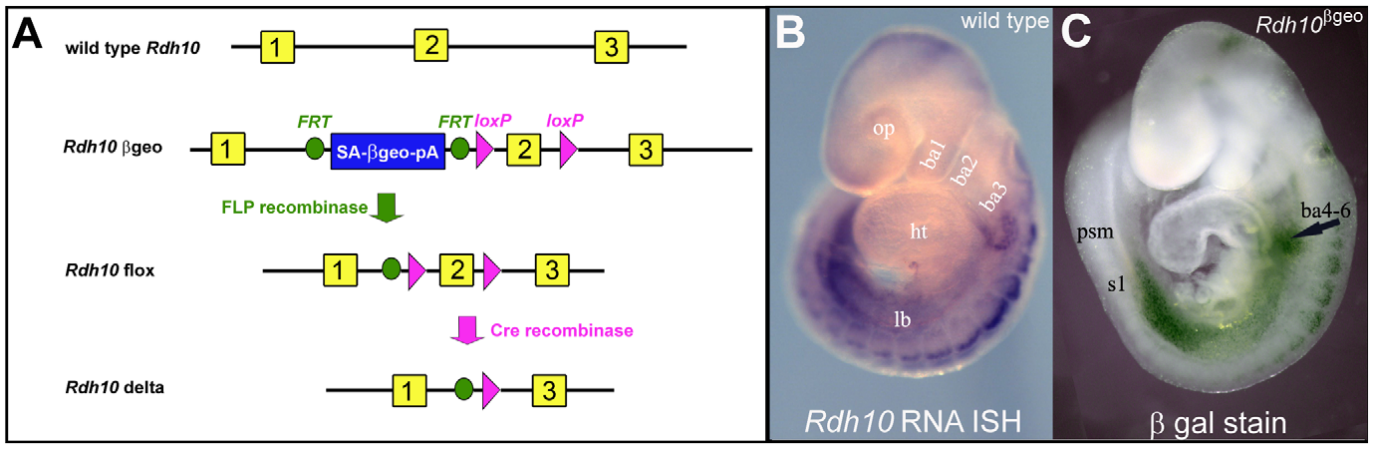
Targeted *Rdh10* gene trap and knockout allele. (A) Schematic diagram of *Rdh10* knockout allele obtained as ES cells from KOMP consortium and derivative alleles. For *Rdh10^βgeo^* allele, a gene-trap splice acceptor *βgeo* cassette is introduced between exon 1 and exon 2 of *Rdh10* and loxP sites are introduced surrounding exon 2. For the *Rdh10^flox^* allele, the gene trap cassette is excised returning function, conditionally, to the *Rdh10* gene. For the *Rdh10^delta^* allele, exon 2 is deleted by Cre excision of the DNA between the loxP sites. (B) *Rdh10* RNA in situ hybridization of E9.5 wild type embryo reveals expression of Rdh10 in specific tissues. *Rdh10* RNA staining pattern n>10 embryos. (C) Staining E9.5 *Rdh10^βgeo^* embryofor β-galactosidase activity (arrow) reveals gene trap transcript expressed in pattern similar to *Rdh10* RNA expression. *Rdh10* β-galactosidase staining pattern n>10 embryos. ba, branchial arch; ht, heart; lb, limb bud; op, optic vesicle; psm, presomitic mesoderm; s, somite.

In order to directly evaluate the contribution of RDH10 in embryonic RA synthesis we sought to completely eliminate RDH10 function from developing mouse embryos. To that end, we generated mice null for RDH10 activity utilizing ES cells produced by the Chori-Sanger-UC Davis (CSD) arm of the international Knockout Mouse Project (KOMP) consortium. The CSD KOMP knockout construct is designed to create a series of alleles that can be generated by sequential deletion through exposure to Cre or FLP recombinase (Fig. 2A). The initial targeted knock-in construct inserts a splice acceptor-lacZ gene trap cassette between exon 1 and exon 2 of the *Rdh10* gene, an allele we call *Rdh10^βgeo^*. The gene trap insertion is expected to produce a null allele by disrupting the endogenous *Rdh10* transcript and producing instead a reporter *lacZ* fusion transcript. Exposure of the gene trap allele to FLP-recombinase can then be used to excise the disruptive splice acceptor cassette (flanked by FRT sites). FLP-mediated excision of the gene trap cassette creates a functionally wild type/conditional knockout allele of *Rdh10* with loxP sites flanking exon 2, an allele we call *Rdh10^flox^*. Cre-mediated recombination can then be used to excise the loxP-flanked exon 2, thereby producing a null allele we call *Rdh10^delta^*. Exon 2 was chosen for deletion because its absence is predicted to create a frameshift mutation thereby truncating any resulting protein product and targeting the transcript for nonsense mediated decay. Prior to distribution, correct targeting of the gene trap construct to the *Rdh10* genomic locus in ES cells was assessed by 5′ and 3′ PCR junction fragment assays at the KOMP production and distribution centers. The quality control tests included long range PCR assays pairing generic targeting cassette primers with *Rdh10* locus-specific genomic primers homologous to the sequences upstream and downstream of the target site.

In order to generate mice bearing an *Rdh10* knockout allele, we obtained from the KOMP consortium ES cell clone Rdh10_D08, which is heterozygous for the *Rdh10* gene trap allele. These ES cells were injected into C57BL6/J blastocysts to produce chimeric mice that were identified by PCR amplification of the *βgeo* fusion gene from tail DNA samples. Male mice chimeric for the *Rdh10* gene trap allele were then mated to identify individual animals transmitting the targeted allele. Mice carrying the *Rdh10^βgeo^* gene trap allele were crossed with mice carrying ubiquitously expressed FLP recombinase [30] to generate a conditional *Rdh10^flox^* allele. Exposure of the gene trap allele to FLP recombinase resulted in excision of the gene trap*/βgeo* portion of the targeting cassette and leaving a pair of loxP sites flanking exon 2 to create the conditional allele. *Rdh10^flox^* mice were then crossed with mice bearing a female germline expressed Cre recombinase [31]. The resulting exposure of the *Rdh10^flox^* allele to Cre recombinase caused excision of the loxP-flanked exon 2 thereby generating *Rdh10^delta^* allele.

Embryos heterozygous for the *Rdh10^βgeo^* gene trap alleles were assessed for expression of the reporter *βgeo* transcript by staining for β-galactosidase activity. In these embryos β-galactosidase activity was very weak but the *Rdh10^βgeo^* gene trap transcript could be detected in a pattern similar to that of the endogenous *Rdh10* transcript (Fig. 2 B–C). Both the endogenous *Rdh10* mRNA, detected by RNA *in situ* hybridization and the *Rdh10^βgeo^* gene trap reporter are present in the posterior pharyngeal region and the lateral plate mesoderm between the fore- and hind-limb buds. Expression of *Rdh10* mRNA and the *Rdh10^βgeo^* gene trap reporter are also present in the isthmus of the midbrain and in the dorsal somites. These sites correspond to the previously reported expression domains for *Rdh10* [23], [32].

### *Rdh10* gene trap allele causes severe retinoid deficiency phenotype

**Figure 3:**
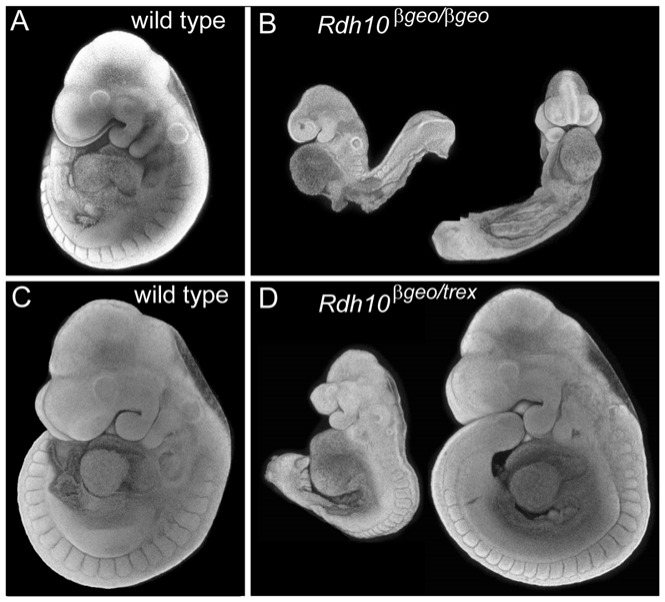
Phenotype of *Rdh10^βgeo^* gene trap allele. (A) Wild type and (B) homozygous *Rdh10^βgeo/βgeo^* embryos were collected at E9.5 (*Rdh10^βgeo/βgeo^* n = 9). Wild type (C) and *Rdh10^βgeo/trex^* compound heterozygous (D) embryos were collected at E9.75 (n = 6). Formalin-fixed embryos were stained with DAPI and imaged by confocal microscopy. For each embryo, a Z-stack of confocal slices was collapsed to form a “pseudo-SEM” image.

Animals heterozygous for the *Rdh10^βgeo^* allele are viable and fertile, with no overt abnormalities. Homozygous *Rdh10^βgeo/βgeo^* embryos, however, exhibit all the hallmarks of extreme retinoid deficiency, with a phenotype remarkably similar to embryos lacking *Aldh1a2*. At E9.5, *Rdh10^βgeo/βgeo^* embryos display defects in embryo growth and axial turning (Fig. 3A–B). Posterior pharyngeal arches are absent. Otic vesicles are present, but reduced in size. Somites are small and extension of the body axis is impaired. Hearts are unlooped and edemic. The parallels between the *Rdh10^βgeo/βgeo^* phenotype and phenotypes of *Aldh1a2^−/−^* mutants indicate that RDH10 deficiency is as deleterious to embryonic growth and patterning as is ALDH1A2 deficiency.

Mice carrying the *Rdh10^βgeo^* allele were crossed with *Rdh10^trex^* to generate compound heterozygote *Rdh10^βgeo^* ^/*trex*^ embryos. The compound heterozygous embryos displayed a range of phenotypes (Fig. 3 C–D). The most severely affected resembled *Rdh10^βgeo/βgeo^* embryos and the mildest were similar to *Rdh10^trex/trex^* embryos. Failure of the *Rdh10^β^* ^geo^ allele to complement the *Rdh10^trex^* mutation provides genetic confirmation that the *Rdh10* conditional construct is correctly targeted to the *Rdh10* locus.

### *Rdh10* deletion allele causes severe retinoid deficiency phenotype

**Figure 4:**
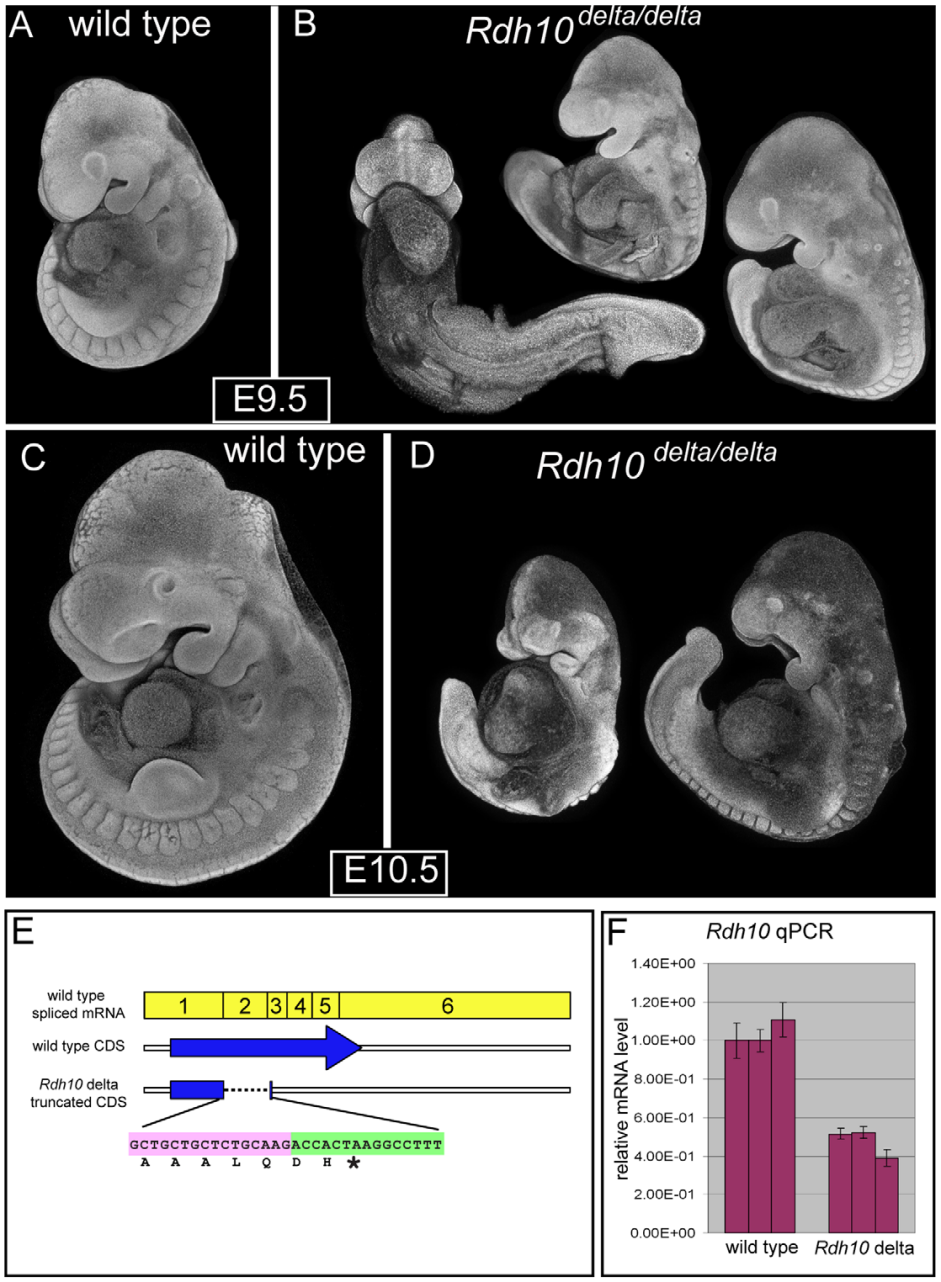
Phenotype and characterization of transcript of *Rdh10^delta^* allele. (A,C) Wild type and (B, D) homozygous *Rdh10^delta/delta^* embryos were collected at E9.5 (A–B) and E10.5 (C–D. (E9.5 mutant embryos n = 34; rightward turn ¾ complete n = 33. E9.5 with leftward turn n = 1. E10.5 mutant embryos n = 5). Formalin-fixed embryos were stained with DAPI and imaged by confocal microscopy. For each embryo, a Z-stack of confocal slices was collapsed to form a “pseudo-SEM” image. (E) Schematic diagram of *Rdh10* spliced mRNA exon structure, along with coding sequence for *Rdh10* wild-type (full length) and *Rdh10^delta^* mutant (truncated) as determined by direct sequencing of reverse transcribed *Rdh10* mRNA. Yellow boxes indicate spliced exons of *Rdh10* mRNA. Dark blue arrow represent coding sequence of spliced wild type *Rdh10* mRNA, blue boxes beneath arrow symbolize truncated coding sequence resulting from splicing of exon 1 directly to exon 3. Nucleotide and protein sequence indicated beneath schematic diagram shows 3′ end of exon 1 spliced directly to 5′ end of exon 3, generating a premature stop codon. Pink box indicates exon 1 derived sequence, green box represents exon 3 derived sequence. (F) Histogram of elative level of *Rdh10* mRNA as assessed by reverse transcription qPCR.

Similar to the *Rdh10^βgeo^* mice, animals heterozygous for the *Rdh10^delta^* mutation are also viable and fertile with no obvious defects. *Rdh10^delta/delta^* embryos can be recovered at the expected Mendelian frequency at E9.5 and E10.5 but viable homozygous embryos are not recovered at later stages (*Rdh10^delta/delta^* genotype: E9.5 n = 12/47, E10.5 n = 4/10, E11.75–E12.5 n = 0/32). In the homozygous state, the *Rdh10^delta^* allele produces all the classic hallmark phenotypes of severe retinoid deficiency (Fig. 4 A–D). *Rdh10^delta/delta^* mutants have defects in growth and axial turning. Axial turning defects are most apparent at E9.5, when many *Rdh10^delta/delta^* embryos appear to be caught in an abnormal leftward “J” shape or, occasionally, a rightward “L” body shape when viewed from the front (Fig. 4 B). Some embryos have a milder phenotype in which the tailbud is positioned in front of the embryo, indicating that axial turning is complete. The growth defect in *Rdh10^delta/delta^* mutants is most obvious at E10.5. For wild type embryos, overall embryo size increases substantially between E9.5 and E10.5 (Fig. 4 A, C). *Rdh10^delta/delta^* embryos, in contrast, appear to cease growth and axis extension around E9.25. *Rdh10^delta/delta^* embryos have abnormalities in many tissues and structures affected by retinoid deficiency including the heart, otic vesicle, pharyngeal arches and forelimb buds. Cardiac defects are observed in all *Rdh10^delta/delta^* embryos. Hearts fail to undergo normal looping and chamber formation, remaining, instead, simple tubes oriented vertically and enlarged by edema. Otic vesicles are present, but are tiny and occasionally duplicated. Pharyngeal arch defects are also apparent in *Rdh10^delta/delta^* embryos. First arches are small and malformed, while arches posterior to the first are absent altogether in most embryos. Forelimb buds are reduced in size or absent.

The *Rdh10^delta^* construct was designed to cause a null mutation via deletion of exon 2, generating a stop codon that should disrupt the open reading frame of the *Rdh10* mRNA and target the transcript for nonsense mediated decay. In order to verify that the *Rdh10^delta^* transcript encodes a premature stop codon, we characterized *Rdh10* cDNA from wild type and *Rdh10^delta/delta^* mutant embryos. The wild type *Rdh10* mRNA encodes an open reading frame that spans the 6 spliced exons of the transcript (Fig. 4 E). Amplification of *Rdh10* cDNA from *Rdh10^delta/delta^* embryos produced a distinct product 235 bp shorter than the wild type cDNA. Direct sequencing of the mutant *Rdh10* cDNA revealed that exon 2 is absent and exon 1 is spliced directly to exon 3, producing a frame shift and generating a stop codon within the open reading frame in the third codon of exon 3 (Fig. 4 E). Protein produced from the *Rdh10^delta^* transcript would be encoded by exon 1 only. Quantitative RT- PCR was performed to assess transcript levels of the *Rdh10^delta^* transcript. Transcript from *Rdh10^delta/delta^* embryos was detected at ∼50% the level produced by wild type embryos, suggesting that the *Rdh10^delta^* transcript is targeted for degradation by nonsense mediated decay (Fig. 4 F).

### Loss of *Rdh10* or *Aldha2* reduces RA production

**Figure 5:**
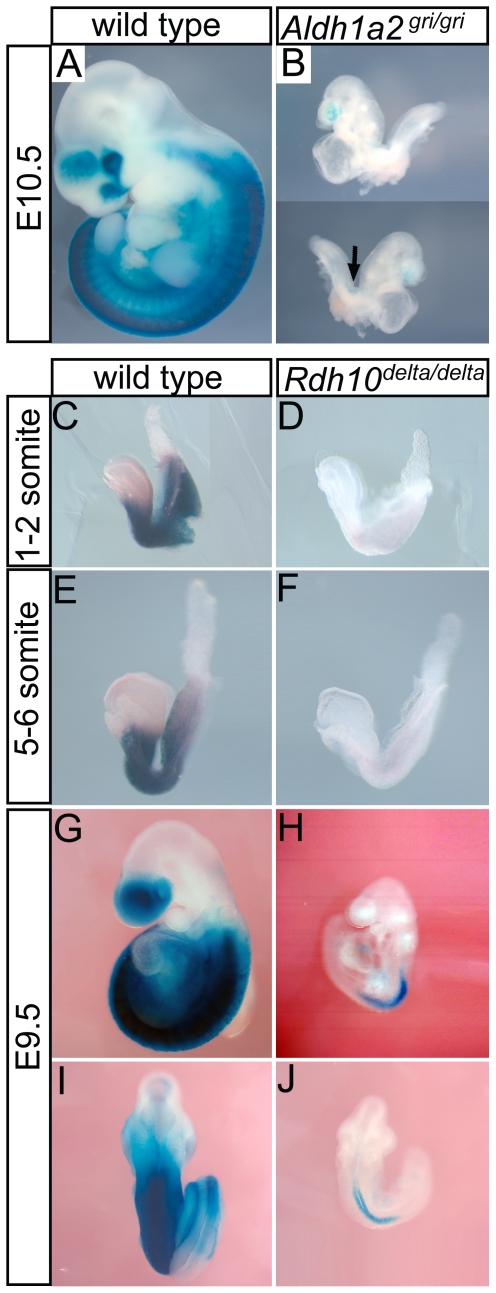
Distribution of RA signaling in *Rdh10* and *Aldh1a2* mutant embryos. Embryos carrying the RARE-lacZ reporter transgene were collected at E8.5, E9.5 or E10.5 and stained for β-galactosidase activity to visualize RA signaling. Blue staining indicates presence of RA. (A–B) *Aldh1a2^gri/gri^* and littermates at E10.5. Arrow indicates small amount of RA signaling detected in neural tube of mutant embryos, n = 13. (C–F) *Rdh10^delta/delta^* and littermates at E8.5, n = 5. (G–J) *Rdh10^delta/delta^* and littermate embryo at E9.5, n = 6. Embryos shown in lateral (G–H) or dorsal (I–J) view.

RDH10 and RALDH2 play sequential roles in synthesis of RA. In order to assess the extent to which the new *Rdh10^delta^* and *Aldh1a2^gri^* mutations disrupt RA synthesis, we utilized the RARE-lacZ reporter transgene, which allows the level and distribution of RA signaling to be visualized by staining for β-galactosidase activity [33]. Loss of RALDH activity in *Aldh1a2^gri/gri^* mutant embryos causes a severe reduction in RA signaling (Fig. 5 A–B), as has been observed for previously reported knockout alleles of *Aldh1a2* [8], [27]. Whereas wild type littermate embryos have strong RA signaling throughout the trunk and within the forebrain, homozygous *Aldh1a2^gri/gri^* mutant embryos are almost completely devoid of RA signaling. A small residual amount of RA signaling can be detected in mutant embryos in the forebrain, consistent with the RA signaling in previously reported alleles [8], [27]. In the mutants we examine here, there is also a very small amount of residual RA signaling detected within the trunk, specifically within the neural tube region at the level of the first few somites. These data demonstrate that the *Aldh1a2^gri^* mutation is either a null allele or extreme hypomorph. The residual RA signaling observed within the forebrain of mutant embryos corresponds to the signal in previously reported knockout alleles of *Raldh2* and likely results from activity mediated by RALDH1 and RALDH3. The small amount of RA signaling detected in the trunk of *Aldh1a2^gri/gri^* mutant embryos, which has also been observed in one of the established *Aldh1a2* knockout alleles [34], may result from a tiny fraction of wild type activity remaining in the *Aldh1a2^gri^* allele or from variation in the null phenotype on different strain backgrounds.

Analogous to the RA attenuation resulting from disruption of *Aldh1a2*, loss of RDH10 function in the *Rdh10^delta/delta^* mutants also elicits a dramatic reduction in RA signaling (Fig. 5 C–J). At E8.5 RA signaling in mutant embryos is essentially absent (Fig. 5 C–F). At E9.5 residual RA signaling can be detected in *Rdh10^delta/delta^*embryos within the trunk neural tube region, the extent and pattern being slightly broader than is seen for the *Aldh1a2^gri/gri^* mutants (Fig. 5 G–J). Residual RA signaling within the forebrain, which is observed in *Aldh1a2^gri/gri^* mutants, is completely abolished in *Rdh10^delta/delta^* embryos. For the *Rdh10^delta/delta^* mutant embryos, as with the *Rdh10^trex/trex^* mutant embryos, the residual RA signaling in the trunk region is localized to the neural tube. *Rdh10^delta/delta^* embryos also retain a small amount of residual signaling in the heart and tissue dorsal to the heart. *Rdh10^delta/delta^* embryos have variable residual RA signaling that is somewhat reflective of their variability in phenotype. These data indicate that, at the critical E8.0–E8.5 stage of development, RDH10 is essential for nearly all RDH activity within the early developing embryo. At E9.5 and later stages, the residual RA detected indicates that other enzymes can contribute a small amount of RDH activity to metabolism of Vitamin A into RA and the small amount of RA produced results in signaling concentrated within the trunk neural tube.

### *Rdh10* transcription is upregulated by retinoid deficiency

**Figure 6:**
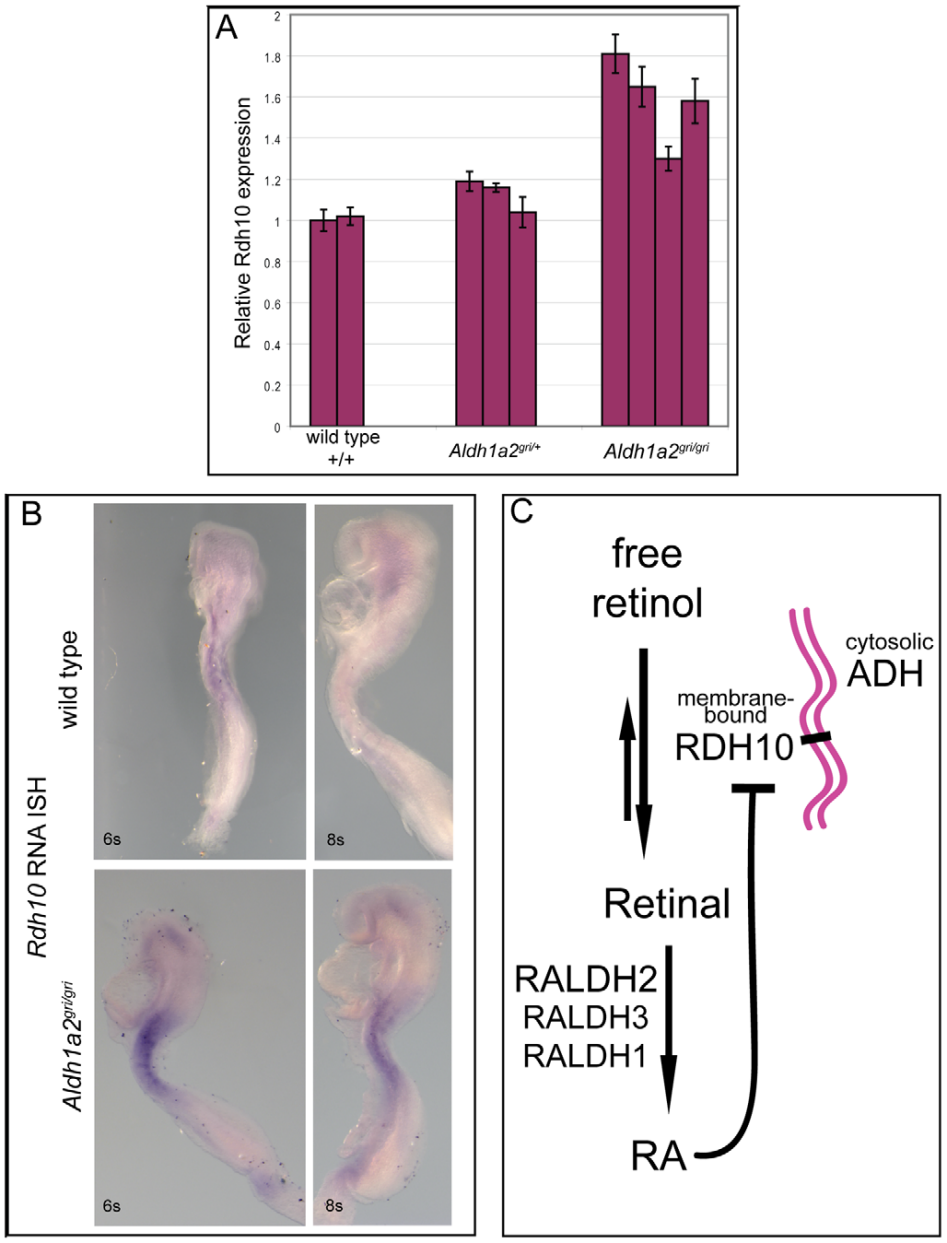
*Rdh10* is up-regulated by reduction of RA. (A) Histogram representing relative levels of *Rdh10* mRNA from wild type, heterozygous *Aldh1a2^gri/+^* and homozygous *Aldh1a2^gri/gri^* embryos, as assessed by quantitative RT-PCR, demonstrating elevated levels of *Rdh10* transcript in homozygous *Aldh1a2^gri/gri^* mutants. (B) RNA *in situ* hybridization for expression of *Rdh10* mRNA in E8.5 wild type and *Aldh1a2^gri/gri^* mutant embryos, demonstrating that the increased *Rdh10* expression occurs in a pattern similar to the normal expression pattern for the gene (mutant embryos n = 5). (C) Model depicting that metabolic product RA feeds-back to reduce expression of membrane bound *Rdh10* in mouse embryos. Pink lines represent that RDH10 and RDH reaction occur in membrane-bound compartment. Cytosolic ADH enzymes do not contribute to any physiologically relevant production of RA in the embryo.

The severe phenotype of the *Rdh10^βgeo/βgeo^* and *Rdh10^delta/delta^* mutants and dramatic reduction in RA synthesis, indicate that RDH10 is responsible for most RDH activity within the early embryo and raises the possibility that the RDH reaction could function as a control point in converting Vitamin A to RA. We therefore sought to determine if embryo RA levels feed back to regulate transcription of the *Rdh10* gene. To that end we evaluated *Rdh10* expression in *Aldh1a2^gri/gri^* mutant embryos in which RA production is severely limited. *Rdh10* expression levels were assessed by quantitative RT-PCR of E9.5 *Aldh1a2^gri/gri^* mutant embryos and their wild type and heterozygous littermates (Fig. 6 A). The RA deficient *Aldh1a2^gri/gri^* embryos have significantly elevated *Rdh10* expression levels relative to wild type embryos, with expression levels in homozygous mutant embryos ranging between 1.3-fold and 1.8-fold higher than wild type and heterozygous littermates. The elevated *Rdh10* transcript in *Aldh1a2^gri/gri^* mutant embryos was expressed in the normal spatial distribution within the embryo at E8.5 (Fig. 6B). These data showing elevation of *Rdh10* expression in RA-deficient *Aldh1a2^gri/gri^* mutant embryos clearly demonstrate that *Rdh10* transcription is regulated by feedback from the metabolic product RA in mice. Interestingly, *Rdh10* contains a potential RAR-RXR (RARE) sequence within exon 5, however to date it is not known whether this is a functional motif nor if it acts in a negative or positive fashion in response to retinoid signaling as has been observed for other RAREs. Nonetheless, our results provide compelling evidence that the RDH reaction is an important control point in regulating metabolism of Vitamin A into RA in mammals and sets the stage for future examination of the complexities of feedback and feedforward enzymatic regulation during this process.

## Discussion

### Importance of RDH reaction in RA production

Vertebrate embryonic development depends upon precise spatial and temporal control of RA signaling. Two sequential enzymatic steps are required for metabolism of retinol into RA, the RDH first reaction and the RALDH second reaction (Fig. 6C). Biochemically, either step could function as a control point for regulating synthesis of RA. However, because the RDH first reaction was initially attributed to ubiquitous or redundant members of the ADH family and was thought to occur in an unregulated fashion, the initial step of Vitamin A metabolism was interpreted as being unimportant for the control of RA synthesis. Over time this led to acceptance of the view that that control of Vitamin A metabolism and RA synthesis was driven predominantly by the RALDH second step.

The view that the RDH first reaction occurs in a ubiquitous and unregulated fashion was challenged by the identification of *Rdh10^trex^*, a mutation that dramatically impairs RA synthesis via disruption of RDH10, a spatially and temporally regulated membrane-bound SDR. However, because the RDH reaction had previously been thought to be carried out by redundant or ubiquitous ADH family members, and because the *Rdh10^trex^* mutant embryos exhibited a milder phenotype and survived longer than embryos lacking *Aldha2*, the RDH first reaction continued to be thought of as relatively unimportant for the spatiotemporal control of RA synthesis. The residual production of RA and the less severe phenotype of *Rdh10^trex^* embryos compared to *Aldh1a2* mutant embryos indicated that some RDH activity remained intact in the *Rdh10^trex^* embryos, mediated possibly by remaining activity of the hypomorphic *Rdh10^trex^* point mutant enzyme, or by other RDH enzymes, or by ADH enzymes. Given that genetic or environmental perturbations causing excessive or insufficient levels of RA cause embryonic malformations, it is important to resolve the question of which enzymes catalyze the RDH reaction during embryogenesis and, further, to understand whether these enzymes function redundantly and ubiquitously or if their regulated expression may serve as a control point for production of RA.

To gain insight into the enzymes and regulation of RA synthesis, we have generated a knockout mutation of *Rdh10* and demonstrate that complete loss of the RDH10 enzyme results in embryonic death ∼E10.5. *Rdh10^delta/delta^* embryos have severe defects in growth, axial turning, and cardiac and pharyngeal development. The severe embryonic phenotypes of *Rdh10^delta/delta^* mutants unequivocally demonstrate that redundant or ubiquitous enzymes, including the members of the ADH family, do not contribute significantly to physiologically relevant production of RA during embryogenesis. Moreover, the severity of the phenotypes resulting from loss of an individual enzyme, RDH10, provides clear proof that the first RDH reaction of Vitamin A metabolism is as critical a control point in the synthesis of RA as the second RALDH reaction.

Although the phenotype of *Rdh10^delta/delta^* mutant embryos is very severe, it is not identical to the extreme phenotype of loss of *Aldha2*. Loss of RALDH2 function results in embryos that apparently cease to grow at by E8.5, whereas loss of *Rdh10* results in embryos that apparently cease to grow at around E9.0. The slightly milder phenotype of the *Rdh10^delta/delta^* embryos is likely due to the small amount of residual RA produced at E9.5 within the trunk region of affected embryos. The identity of the enzymes responsible for the residual trunk RA at E9.5 is unknown. It is possible the small amount of residual RA production in *Rdh10^delta/delta^*mutant embryos represents the contribution of other, as yet unidentified, SDR enzymes closely related to RDH10 or, alternatively, ADH enzymes. Recently, we demonstrated that a compound knockout of multiple *Adh* genes does not impair the residual RA synthesis in *Rdh10^trex^* embryos nor exacerbate the phenotype [35], a result which argues against a role for ADHs. Whichever enzymes are ultimately found to be responsible for the small amount of residual RA, it is clear that they are insufficient to produce physiologically relevant levels of RA and are unable to compensate for loss of *Rdh10*.

Concurrent with the present study, a distinct knockout allele of *Rdh10*, a deletion of *Rdh10* exon 4, was reported in the literature [36]. The phenotypes of the *Rdh10* exon 4 deletion allele (*Rdh10^−/−^*)are largely consistent with the *Rdh10^delta^* allele we describe here. In both cases, the majority of embryos survive until ∼E10.5 and exhibit defects in growth and extension of the body axis, absence of posterior pharyngeal arches, compacted somites, and small or duplicated otic vesicles. RA signaling is also notably absent from the forebrain region in both knockout models at this stage. The primary difference is that we do not observe embryo survival beyond E10.5 in our *Rdh10^delta^* mutants, whereas a small fraction (∼10%) of exon 4 deletion mutants survive until E12.5. This difference may be related to variation in strain background or the relative importance of the individual exons for Rdh10 activity that were deleted. We also examined RA signaling in *Rdh10^delta/delta^* embryos in a broader range of embryos and observe that between the 1–6 somite stages (Fig. 5C–F), RA signaling is not detected in *Rdh10^delta/delta^* embryos compared to the robust detection of reporter activity in control embryos. Taken together, these data indicate the important role of *Rdh10* in RA signaling during the early critical E8.0–8.5 window of development.

### Feedback regulation on RA metabolism

In addition to showing that RDH10 is the predominant enzyme responsible for embryonic conversion of retinol to retinal, the data presented here reveal that *Rdh10* gene expression is modulated by presence or absence of RA, being upregulated by RA deficiency in *Aldha2^gri/gri^* mutants. The upregulation of *Rdh10* expression when RA synthesis is impaired demonstrates that *Rdh10* is a point for feedback control in Vitamin A metabolism and RA synthesis in mammals (Fig. 6C). A similar mode of regulation has been observed in *Xenopus* and zebrafish, indicating that the mechanistic feedback control of *Rdh10* to modulate the RA synthesis pathway is evolutionarily conserved [24], [25]. The evolutionary conservation of feedback regulation of the *Rdh10* gene by RA lends further support to the concept that RDH10-mediated oxidation of retinol to retinal plays an important role in regulating the key process of embryonic Vitamin A metabolism and RA synthesis.

The conversion of retinol to retinal is a reversible reaction. Enzymes of the SDR family can mediate both the forward and the reverse directions. Transcriptional regulation of SDR enzymes to mediate RA homeostasis is observed in other contexts. In zebrafish embryos, excess RA induces expression of *dhrs3a*, an SDR that catalyzes the reduction of retinal into retinol [25]. In adult mouse liver, chronic exposure to ethanol causes complex changes in RA metabolism and is associated with transcriptional changes in RA metabolic genes including induction of the SDR *Dhrs9* [37]. Together these data suggest that reversible interconversion of retinol to retinal by SDRs serve as a key control step in RA homeostasis. Consistent with this, biochemical analysis of Vitamin A metabolism in embryos has indicated that the first enzymatic reaction, the oxidation of retinol to retinal, is the rate-limiting step in the synthesis of retinoic acid [38].

### Spatial localization of RA production in tissue and cell

The finding that the RDH reaction, mediated primarily by RDH10, is a major control point in RA synthesis has implications for understanding the tissue specificity and sub-cellular localization of the sequential steps of RA production. With respect to tissue distribution of Vitamin A metabolism and RA synthesis, it is notable that the expression pattern of *Rdh10* within an embryo is very similar to, but not identical to that of *Aldh1a2* [23], [32]. In chick, the expression pattern of *Rdh10* has also been shown to overlap with that of the gene encoding the retinol transporter *Stra6* [26]. Together, these data suggest that retinol is imported into cells, oxidized into retinal and subsequently into the product RA all largely within the same population of cells. We have previously shown that maternal administration of intermediate retinal, which exposes all cells of the embryo to the retinal intermediate, can rescue RDH10 deficient embryos to birth [39] and recently this has now been extended postnataly [36]. Although viable, these rescued embryos still exhibit some abnormalities including limb and behavioral defects. The defects remaining in retinal rescued animals indicate that while embryo viability is not dependent on spatially restricted retinal production, proper tissue patterning and development are.

With respect to sub-cellular localization of Vitamin A metabolism, the medium chain reductase ADH enzymes are known to function within the cytosol, while the SDR RDHs function in a membrane bound capacity. RDH10 is associated with the membrane-bound compartment [40] and its membrane association is necessary for its function [41]. Recently, we demonstrated that the RDH reaction occurs not in the cytosol where ADHs reside, but in a membrane-bound cellular compartment such as that occupied by RDH10 [39]. Within the cytosol, RBP1 binds to retinol and inhibits oxidation. The membrane localization of RDH10 is functionally important, presumably because it allows RDH10 to oxidize RBP1-free pools of retinol available in the membrane compartment (Fig. 6C). These findings suggest that ADHs do not contribute significantly to retinol oxidation because they cannot oxidize the RBP1-bound retinol in the cytosol and are unable to access RBP1-free retinol from membranes (Fig. 6C). They explain why the cytosolic ADHs, although widely expressed, cannot compensate for the loss of RDH10 during embryogenesis.

The lack of function of ADHs in embryonic retinol oxidation is consistent with studies of individual and compound *Adh* knockout mice, which suggest that ADHs function primarily in governing post-natal retinoid homeostasis (reviewed in [35]). In adult mice, ADH1 provides considerable protection against vitamin A toxicity [42], [43] whereas ADH4 promotes survival during vitamin A deficiency [44], a difference which illustrates distinct physiological functions for these two enzymes in retinol metabolism. In contrast, ADH3 appears to function redundantly in both the prevention of retinol toxicity and Vitamin A deficiency, a dual role consistent with its ubiquitous expression. Collectively, these biochemical and compound mutant analyses strongly argue against a significant role for ADHs in Vitamin A metabolism and RA synthesis during embryonic development.

Given that *Rdh10* is the predominant enzyme oxidizing retinol to retinal and the mounting evidence that ADHs make little if any contribution, we conclude that the conversion of retinol to retinal within an embryo must occur predominantly, if not completely, within a membrane-bound compartment. Collectively these data demonstrate the critical roles played by *Rdh10* in mediating Vitamin A metabolism and synthesis of RA and as a nodal point in governing the feedback regulation of RA production. Considering the relatively discrete sites of *Rdh10* expression, the allelic series of *Rdh10* alleles including conditional alleles described here, will facilitate important strategies to characterize the consequences of impaired Vitamin A metabolism and RA synthesis in a precisely controlled spatiotemporal manner.

## Materials and Methods

### Generation of *Rdh10* conditional mice

The *Rdh10* mouse strains used for this research project were generated from ES cells obtained from the trans-NIH Knockout Mouse Project (KOMP) Repository, a NCRR-NIH supported strain repository. ES cells were created by CSD consortium from funds provided by the KOMP. To inquire about KOMP products go to www.komp.org or email service@komp.org. The Rdh10_D08 (full clone name, EPD0149_1_D08) ES cell clone was produced by the CSD consortium (Childrens Hospital of Oakland Research Institute, The Sanger Institute, and UC Davis). Quality control for correct targeting was assessed by the KOMP production and distribution centers using PCR based assays that spanned the 5′ and 3′ junction fragments. The parental ES cell line was JM8.N4, which is a C57BL/6N background. For the mice described in this report, we obtained Rdh10_D08 ES cells from KOMP and injected them into C57BL/6 blastocysts. The *Rdh10* gene trap line was thereby established and subsequently maintained on a C57BL6/J background. Some animals, such as those crossed with transgenic reporter lines, were mixed with other backgrounds. The KOMP repository independently generated mice from the same clone of ES cells. The strain name for the KOMP generated mice is C57BL/6N-Rdh10^tm1a(KOMP)Wtsi^.

*Aldh1a2^gri^* mutant mice were originally described as *grimace* mutants obtained as part of a chemical mutagenesis screen for defects in early craniofacial development [29]. Mice carrying the FLP recombinase (FLPeR) in the Rosa26 locus on a C57BL6 background [30] available from the Jackson Laboratory as stock #009086 B6.129S4-*Gt(ROSA)26Sor^tm1(FLP1)Dym^*/RainJ. Mice carrying a Cre recombinase transgene expressed in oocytes driven by ZP3 promoter sequences [45] are available from the Jackson Laboratory as stock #003651 C57BL/6-TgN(Zp3-Cre)93Knw.

### Genotyping

All genotyping was accomplished by real-time PCR assays performed by the commercial genotyping service Transnetyx (Transnetyx.com). For the wild type *Rdh10* allele assay, a probe denoted “Rdh10-1 WT” was used to detect a fragment spanning the region of the unmodified genomic locus corresponding to the insertion site of the knock in construct. For the *Rdh10^βgeo^* allele, an assay was used to detect the presence of lacZ and neomycin, corresponding to the *βgeo* cassette. For the *Rdh10^delta^* allele, a probe denoted “Rdh10-1EX” was used, which detects an amplified region spanning the junction fragment covering the single recombined FRT site and the 3′ loxP site. To test for homozygosity of the null allele, samples were screened for absence of *Rdh10* exon 2 using an assay probe designated “Rdh10-2 WT”. The following sequences indicate the relevant amplified regions, excluding primers:

*Rdh10* wild type allele

GCTCATTCATTGCTTGGCTTTGAATGAGTTTAGATTGTTGCTTACAGTTTGGATTTGTGAGCCTTAAAGTGAATTTC

*Rdh10^delta^* allele

AGTTCCTATTCCGAAGTTCCTATTCTCTAGAAAGTATAGGAACTTCGTCGAGATAACTTCGTATAGCATACATTATACGAAGTTATGGTCTGAGCTCGCC

### qPCR

To assay for Aldh1a2 expression in *Aldh1a2^gri^* mutant embryos, qRT-PCR TaqMan assay was used with the following probe and primers:

Aldh1a2Fwd-TGA TAA AAT TCA CGG ATT GAC CAT

Aldh1a2Rev-GGG CTC GTG TCT TGT GAA AGT AA

Aldh1a2TaqMan Probe - CCTGTAGATGGAGACTAT

To assay for *Rdh10* expression in wild type and *Aldh1a2^gri^* mutant embryos, qRT-PCR SYBR assay was used with the following primers:

Rdh10-F: TGG TGT GGT TTC TGG ACA TCA CCT

Rdh10-R: CTC TAG CAT CGT TGG AAG AAA GGC

To assay for altered *Rdh10* mRNA in *Rdh10^delta^* mutant embryos, qRT-PCR SYBR assay was used with the following primers:

Rdh_q1F: CTGGGACTGTTCAGCACTG

Rdh_q1R: TCTACAAGGTAAGGGCAAACC

### RNA *in situ* hybridization and pseudo-SEM imaging of whole mouse embryos

Whole mount *in situ* hybridization for *Rdh10* was performed as in [23]. Embryo morphology was visualized by staining whole embryos with DAPI to label all cell nuclei and imaging fluorescent signal. Embryos were fixed in 4% formalin overnight at 4°C then stained with 2 ug/ml DAPI in PBS and imaged by confocal microscopy using an LSM5 Pascal confocal microscope. For each embryo, a Z-stack of confocal slices was collapsed to form a “pseudo-SEM” image. RA signaling was visualized by staining embryos carrying the RARE-lacZ transgene for β-galactosidase activity. A subset of the brightfield embryo images (Fig. 2D, Fig. 5 G–J, 6B) were processed with Helicon Focus image processing software to compile and render the focused regions of multiple focal planes as a single in-focus image. A minimum of 5 embryos were used for each parameter analysed unless otherwise stated.

### Web addresses for reagents and technique resources

Transnetyx automated qPCR genotyping: www.Transnetyx.com

Knockout Mouse Project: www.KOMP.org

Rdh10 conditional knockout vector: http://www.sanger.ac.uk/htgt/report/project_gene_report?project_id=34040
